# Characterization of Extended-Spectrum Cephalosporin (ESC) Resistance in *Salmonella* Isolated from Chicken and Identification of High Frequency Transfer of *bla*_CMY-2_ Gene Harboring Plasmid In Vitro and In Vivo

**DOI:** 10.3390/ani11061778

**Published:** 2021-06-14

**Authors:** Bo-Ram Kwon, Bai Wei, Se-Yeoun Cha, Ke Shang, Jun-Feng Zhang, Hyung-Kwan Jang, Min Kang

**Affiliations:** 1 Department of Veterinary Infectious Diseases and Avian Diseases, College of Veterinary Medicine and Center for Poultry Diseases Control, Jeonbuk National University, Iksan 54596, Korea; aniscikwon@gmail.com (B.-R.K.); weibai116@hotmail.com (B.W.); kshmnk@hanmail.net (S.-Y.C.); shangke0624@gmail.com (K.S.); jfzhang018@gmail.com (J.-F.Z.); 2 Bio Disease Control (BIOD) Co., Ltd., Iksan 54596, Korea

**Keywords:** *Salmonella*, chicken, extended-spectrum cephalosporin, *bla*_CMY-2_, mouse, frequent transfer

## Abstract

**Simple Summary:**

The prevalence of extended-spectrum cephalosporin (ESC)-resistant *Salmonella* is of great concern, as these strains with the same *β*-lactamase (*bla*) genes were found in human and poultry. The objective is to characterize ESC-resistant *Salmonella* isolated from chicken and to determine the transferability of *β*-lactamase gene-harboring plasmid in vitro and in vivo. ESC resistance genes in *Salmonella* isolated from chickens and presented a comprehensive analysis of the highly frequent transfer of the *bla*_CMY-2_ gene in vitro and in vivo. In addition, this study has demonstrated the ease with which a *bla*_CMY-2_ gene-harboring plasmid can be rapidly transferred between *Salmonella* and pathogenic *E. coli* within the intestinal tracts of mice, even without antimicrobial selective pressure. Given the potential risk of the frequent transfer of the *bla*_CMY-2_ gene via the food chain to the human digestive tract, the molecular mechanism involved in the dissemination and maintenance of ESC resistance genes should be studied as further research in greater detail, and enhanced surveillance should be implemented to prevent the widespread of ESC resistant strains.

**Abstract:**

A total of 136 *Salmonella* isolates from chicken feces and meat samples of the top 12 integrated chicken production companies throughout Korea were collected. Among the 17 ESC-resistant *Salmonella*; *bla*_CTX-M-15_ was the most prevalent gene and two strains carried *bla*_TEM-1_/*bla*_CTX-M-15_ and *bla*_CMY-2_, respectively. The transferable *bla*_CTX-M-15_ gene was carried by IncFII plasmid in three isolates and the *bla*_CMY-2_ gene carried by IncI1 plasmid in one isolate. *bla*_CMY-2_ gene-harboring strain was selected as the donor based on the high frequency of *bla*_CMY-2_ gene transfer in vitro and its transfer frequencies were determined at 10^−3^ transconjugants per recipient. The transfer of *bla*_CMY-2_ gene-harboring plasmid derived from chicken isolate into a human pathogen; enteroinvasive *Escherichia coli* (EIEC), presented in mouse intestine with about 10^−1^ transfer frequency without selective pressure. From the competition experiment; *bla*_CMY-2_ gene-harboring transconjugant showed variable fitness burden depends on the parent strains. Our study demonstrated direct evidence that the *bla*_CMY-2_ gene harboring *Salmonella* from chicken could frequently transfer its ESC-resistant gene to *E. coli* in a mouse intestine without antimicrobial pressure; resulting in the emergence of multidrug resistance in potentially virulent EIEC isolates of significance to human health; which can increase the risk of therapeutic inadequacy or failures.

## 1. Introduction

Recently, an increasing occurrence of extended-spectrum cephalosporins (ESC)-resistant strains has been recognized as a serious threat to human health [1]. Resistance to *β*-lactam antimicrobials is mainly caused by the production of antimicrobial inactivation enzymes called *β*-lactamases [2]. Extended-spectrum *β*-lactamases (ESBL) and AmpC *β*-lactamase (AmpC) are the major *β*-lactamases detected in ESC-resistant strains worldwide [3]. These enzymes are frequently encoded by genes that are located on a plasmid, which is a mobile genetic element that can transfer horizontally within and between different bacterial species [4]. Various studies have suggested that food-producing animals as a reservoir for ESBL/AmpC-producing strains that could promote the transmission of resistance determinants to humans [2]. Similar or identical ESC-resistant isolates or ESBL/AmpC plasmids were found in chicken meat and patients, suggesting poultry and poultry products play a pivotal role in the spread of ESC resistance genes to humans [2].

The fact that the same plasmid is observed in several bacterial strains isolated from poultry and humans confirms that antimicrobial resistance genes can be transferred from poultry to humans [2]. A previous study observed the possibility using in vitro human gut simulation model that there is a transfer from food-borne ESC resistant isolates to other commensal and pathogenic bacteria [5]. However, there is a lack of actual evidence that ESC resistance genes and particularly the *bla*_CMY-2_ gene transfer from poultry to human-origin pathogenic isolates in vivo could cause considerable risks, such as the high possibility of inadequate treatment or therapeutic failures. Antimicrobial resistance, by the acquisition of a mobile genetic element or by mutation, is generally thought to induce a competitive fitness disadvantage on host bacteria in the absence of selective pressure for resistance phenotypes [6]. However, few studies have examined the fitness advantage of their host bacteria after acquired resistance plasmids [7].

This study aimed to clarify the characteristic of ESC-resistant *Salmonella* isolated from chicken and to determine the transferability of ESC resistance-determining plasmid in vitro and in vivo. We also examined the ability to donate ESC resistance genes and how frequently they are transferred from chicken isolates to human pathogens in the mammalian mouse intestine. As antimicrobial resistance is a widely acknowledged factor affecting plasmid persistence in the absence of selective pressure [8], we attempted to identify the contribution of ESC-resistant plasmid in in vitro fitness by competition between susceptible and resistant isolates. Our goals were to assess the interspecific horizontal gene transfer (HGT) of ESC resistance from animal-derived *Salmonella* to human-derived bacteria in vitro and in vivo, also to evaluate the impact of ESC resistance genes acquisition on bacteria fitness.

## 2. Materials and Methods

### 2.1. Bacterial Isolates

A total of 136 *Salmonella* isolates isolated from chicken feces and chicken meat samples from 2017 to 2018 were collected from the top 12 integrated chicken production companies throughout Korea. The isolation and serotyping of *Salmonella* were conducted as described previously [9]. Among 136 *Salmonella* isolates, those showing either ESBL or AmpC phenotype were used in this study. *Salmonella* strains that are resistant to ceftiofur are considered ESBL/AmpC-producing strains. To select a recipient for the in vivo transfer experiment, we obtained a total of 10 strains (Table S1), which were isolated from human patient’s stool samples and categorized as pathogenic *Escherichia coli*, from the National Culture Collection for Pathogens (NCCP) South Korea.

### 2.2. Antimicrobial Susceptibility Test

The antimicrobial susceptibility of all isolates was evaluated by the minimum inhibitory concentrations (MICs) of the test antimicrobial agents amoxicillin/clavulanic acid (AMC), cefoxitin (FOX), cefepime (FEP), ceftazidime (TAZ), ceftiofur (XNL), trimethoprim/sulfamethoxazole (SXT), sulfisoxazole (FIS), chloramphenicol (CHL), ampicillin (AMP), ciprofloxacin (CIP), nalidixic acid (NAL), streptomycin (STR), gentamicin (GEN), tetracycline (TET), meropenem (MERO), and colistin (COL) using the KRNV5F (TREK Diagnostic Systems, Korea). *Escherichia coli* ATCC 25922 was used as the reference strain for quality control. The susceptibility breakpoints of most antimicrobials were interpreted according to the CLSI guidelines [10]. Since CLSI breakpoints were not available for colistin, ceftiofur, and streptomycin, MICs were determined according to the European Committee on Antimicrobial Susceptibility Testing (EUCAST) guidelines [11] for colistin and to Centers for Disease Control and Prevention [12] for the ceftiofur and streptomycin.

### 2.3. Identification of β-Lactamases

After phenotypic screening, PCR were implemented regarding ceftiofur resistant isolates for detecting the presence of *β*-lactamase genes encoding CTX-M, TEM, and CMY-type following a previous protocol [13,14]. Genomic DNA templates for PCR were prepared using fresh *Salmonella* colonies on MacConkey agar (Difco laboratories, Sparks, Maryland, USA) plates by adding 100 µL of sterile distilled water and boiling in a heater block at 100 °C for 15 min. The sequencing reactions were performed by an external company (Solgent, Daejeon, Korea). The obtained amino acid sequences were compared with those in the GenBank nucleotide database using the BLAST online service, provided by the National Center for Biotechnology Information (www.ncbi.nlm.nih.gov/BLAST, accessed on 21 March 2020), to determine the specific types of *β*-lactamase genes.

### 2.4. Plasmid Replicon Typing

Plasmid DNA was extracted using HiYield™ Plasmid Mini Kit (RBC Bioscience, Taipei, Taiwan) according to the manufacturer’s instructions. Plasmid incompatibility groups were determined by the PCR-based replicon typing (PBRT) method [4]. For plasmids such as IncF, IncI1, IncHI2, and IncHI plasmids were subtyped by plasmid MLST (pMLST) (http://pubmlst.org/plasmid/, accessed on 10 April 2020).

### 2.5. In Vitro Conjugation Experiment

A conjugation experiment was performed according to previously reported methods with some modification [15]. In vitro mating was performed in liquid media, and cephalosporin-susceptible *Escherichia coli* J53 (sodium azide-resistance) and selected *E. coli* NCCP isolate (certain antimicrobial resistance) were used as recipients. Briefly, overnight cultures of donor and recipient strains were re-cultured in logarithmic phase (OD 600 nm of 0.5) at 37 °C in fresh tryptic soy broth (Difco Laboratories, Detroit, MI, USA) medium for 4 h. Next, 1 mL of the donor and 4 mL of the recipient were mixed and incubated without shaking for 1 h at 37 °C. The culture was spread on MacConkey agar plates containing sodium azide (200 μg/mL) and ceftiofur (8 μg/mL) for detecting *E. coli* J53-derived transconjugants. MacConkey agar plates containing certain antimicrobial and ceftiofur (8 μg/mL) were used for detecting *E. coli* NCCP isolate-derived transconjugants. The experiment was repeated three times, and three putative transconjugant colonies were randomly selected from each experiment. For verifying the transconjugant, the transconjugant was evaluated by the MICs with the method described above, and the presence of a marker gene of an ESC-resistant plasmid was confirmed by PCR, as previously described [14]. Conjugation frequency was calculated as the ratio of the number of transconjugants per recipient (Tc/R). Recipient isolate counts were calculated by subtracting transconjugant colony counts from the number of colonies obtained on agar plates, which included both recipients and transconjugants.

### 2.6. In Vivo Transfer Experiment

When selecting the recipient for the transfer experiment in vivo, one recipient for the in vivo transfer experiment was selected based on the results of the conjugation frequency test. Enteroinvasive *E. coli* (EIEC) NCCP 13719 carried the virulence gene of *ipa*H [16], which was ceftiofur-susceptible but tetracycline-resistant and showed the highest frequency of transfer, was selected as the recipient for the in vivo transfer experiment.

**Figure 1:**
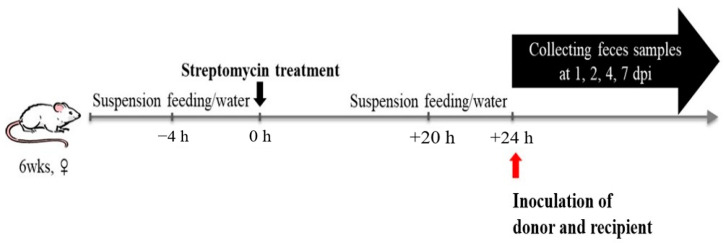
Schematic representation of in vivo transfer experiment set up.

The animal experiment was conducted in accordance with the requirements of the Animal Care and Ethics Committees of Jeonbuk National University and were approved by the National Association of Laboratory Animal Care (JBNU 2021-06). Female SPF 6-week-old BALB/c mice (Samtako, Osan, Korea) were randomly housed in four groups of five animals each, and each group was kept in a separated isolator (Three-Shine, Daejeon, Korea). Before the inoculation of donor and recipient, fecal samples from all mice in each group were pooled, and the absence of resistant strains was confirmed by spreading onto a plate that we used in this study. In addition, fecal samples were also checked to be free of the *bla*CMY-2 gene and *ipa*H gene. The experimental groups were as follows: streptomycin-treated control group (G1), streptomycin-treated and then donor-inoculated group (G2) for monitoring donor strain colonization, streptomycin-treated and then recipient-inoculated group (G3) for monitoring recipient strain colonization, and streptomycin-treated and then donor and recipient simultaneously inoculated group (G4). Before inoculation, streptomycin at a dose of 20 mg per mouse was pretreated to eliminate the circumstance of microbial competition and induce the colonization of inoculating isolates (Figure 1) [17]. Food and water were discontinued 4 h before oral administration of streptomycin. Then, food and water were made available to be consumed ad libitum. At 20 h after streptomycin administration, food and water were ceased again for 4 h before the mice were inoculated orally by gavage with 0.2 mL of 10^8^ CFU/mL of donor and recipient. As for G4, the recipient was inoculated 30 min after inoculating the donor. Then, water was offered immediately and food was made available 2 h after infection ad libitum. On 1, 2, 4, and 7 days after infection, fresh fecal samples were collected from each mouse. The samples were weighed and diluted five-fold and then finally homogenized by vortexing in phosphate buffer saline (PBS). For colony counting, 10-fold serially diluted samples were inoculated onto appropriate agar plates, including antimicrobials for each group. For verification of transconjugant isolates, putative colonies were sub-cultured onto antimicrobial selective agar plates, and genomic DNA was extracted according to the boiling method as described above for using PCR analysis to test for the possession of marker gene (*β*-lactamase gene from donor and virulence gene from the selected recipient). As for G4, transfer frequency was calculated as the ratio of the number of transconjugants per recipient (Tc/R).

### 2.7. Competition Experiment In Vitro

To assess the fitness effect of resistance plasmid carriage, competition assays between resistance plasmid-harboring transconjugant and its parental isolates, *E. coli* J53 and *E. coli* NCCP 13719, were conducted. The competition experiment was carried out as previously described [18] and repeated five times. Briefly, parental isolates were incubated overnight at 37 °C with shaking at 200× *g* rpm in 10 mL of lysogeny broth (LB). Transconjugants were cultured in LB with the addition of 8 μg/mL ceftiofur to ensure the expression of ESC-resistant genes. Overnight cultures of two pairwise strains (*Escherichia coli* J53/transconjugant of *E. coli* J53, *E. coli* NCCP 13719/transconjugant of *E. coli* NCCP 13719) were adjusted to OD 600 nm of 0.5, were diluted 10^−4^, and were then mixed 1:1 in LB broth at 0 h. After 24 h of incubation at 37 °C, the mixed isolates were again 10^−4^ diluted into a fresh LB medium. This procedure was repeated every 24 h until the competition experiment had lasted for 72 h. The total number of isolates were determined by dropping 10 μL of properly diluted samples onto antimicrobial-free and antimicrobial-supplemented selective MacConkey agar plates in triplicate at 24 h, 48 h, and 72 h. The number of CFUs growing on the MacConkey agar plate including ceftiofur (8 μg/mL) was subtracted from the number of CFUs growing on the antimicrobial-free plate to determine the number of ESC-resistant gene-free isolates in the mixed population. To assess the relative fitness of transconjugants compared with its parental strains, an in vitro competitive ratio was calculated using a previously described method [17]. The competitive ratio was defined as the ratio of the number of CFU of the transconjugants vs. the parental strain at 24, 48, and 72 h.

## 3. Results

### 3.1. Characterization of Bacterial Strains

Based on the results of antimicrobial susceptibility assessment of 136 isolates, 17 out of 136 (12.5%) *Salmonella* spp. were consistent with an ESBL/AmpC phenotype and genotype (Table 1). The frequency of ESBL *β*-lactamase production with CTX-M gene was 11.8% (16/136) in *Salmonella* isolates, which was significantly (*p* < 0.05) higher than AmpC *β*-lactamase production (0.7%, 1/136) with CMY gene in *Salmonella* isolates. The four serovars isolated were *Salmonella* Enteritidis (52.9%), *Salmonella* Virchow (35.5%), *Salmonella* Albany (5.9%), and *Salmonella* spp. (5.9%). CTX-M (94.1%) was the most commonly detected *β*-lactamase family, and *S. enteritidis* had CTX-M and TEM gene combination while *S.* Albany was CMY-positive. All strains showed multidrug resistance. For all 17 strains, a conjugation experiment was implemented regarding the transfer of the ESC-resistant gene, and seven (41.2%) out of 17 strains were successfully conjugated in a wide range of frequencies from <10^−7^ to ≥10^−3^ (transconjugant/recipient). Among the transconjugants, one strain, which the harbored *bla*_CMY-2_ gene, showed high transfer frequency (≥10^−^^3^) [19,20]. On analysis using PCR-based replicon typing for conjugative plasmids, IncFIIS plasmids harboring CTX-M-15 were found in three *S.* Enteritidis isolates, and IncI1 plasmid harboring CMY-2 was found in one *S.* Albany isolate. IncI1 plasmids were further submitted to pMLST and assigned to a sequence type of ST12. The remaining three isolates were indicated as non-typeable plasmids.

### 3.2. In Vitro Transfer

Using the liquid mating method, transconjugant with its parental strains, *E. coli* J53 (Tc.J53), and *E. coli* NCCP 13719 (Tc.13719) displayed ESC resistance profile corresponding to the acquisition of the *bla*_CMY-2_ gene (Table 2). Tc.J53 and Tc.13719 expressing of *the bla*_CMY-2_ gene were resistant to ampicillin, cefoxitin, ceftiofur, ceftazidime, and amoxicillin/clavulanic acid (AMC), however, remind to susceptive to fourth-generation cephalosporins of cefepime, which is consistent with an AmpC phenotype. The *bla*_CMY-2_ carrying transconjugants of Tc.J53 did not show any resistance to non–*β*-lactam antibiotics, suggesting that no other resistance genes were located on this IncI1 plasmid.

The conjugation frequency of *bla*_CMY-2_ IncI1 plasmid from *Salmonella* to *E. coli* J53 and *E. coli* NCCP 13719 was determined to be 1 × 10^−3^ ± 1 × 10^−7^ and 2 × 10^−3^ ± 1 × 10^−3^ on an agar plate with 200 μg/mL sodium azide and 8 μg/mL ceftiofur, 100 μg/mL tetracycline and 8 μg/mL ceftiofur, respectively.

### 3.3. In Vivo Transfer

**Figure 2:**
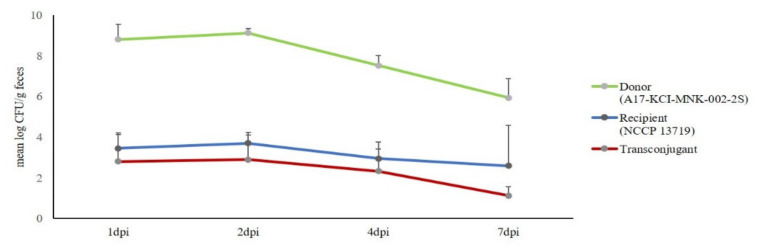
Bacterial counts of the donor, recipient, and transconjugant from mouse fecal samples in group 4 (G4), expressed as the log number of CFU per gram of feces.

To get the information on the efficiency of bacterial intergenic plasmid transfer in the mammalian intestine and to better mimic the in vivo situation, streptomycin-treated mice were used in this study. One-day after inoculation with donor and recipient (G4), the concentration of donor from each mouse ranged from 10^7^ CFU/g to 10^9^ CFU/g and that of recipients ranged from 10^2^ CFU/g to 10^4^ CFU/g (Figure 2 and Figure S1). The frequency of plasmid transfer at 1-day post-infection (dpi) from G4 was estimated at an average of 2 × 10^−1^ ± 4 × 10^−1^ with the ratio of transconjugants per recipient. This showed that the *bla*_CMY-2_ IncI1 plasmid was indeed efficiently transferred from the *Salmonella* isolate to the EIEC in the gut of streptomycin-treated mice. The number of transconjugants did not reach detectable levels at 7 dpi in four out of five mice.

### 3.4. Fitness Cost Assessment by Competition Experiment

**Figure 3:**
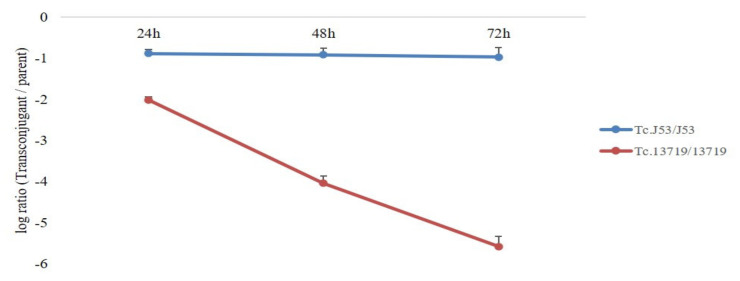
Competitive growth kinetics. Dynamics of replicate competition experiments for parent strains, *E. coli* J53 and *E. coli* NCCP 13719, and their transconjugant containing the *bla*_CMY-2_ gene.

The impact of harboring the *bla*_CMY-2_ gene on host fitness was evaluated by a pairwise competition experiment (Figure 3). Our results showed that the ceftiofur-sensitive strains were out-competing resistant strains in the absence of selective pressure with the value of transconjugant per parent strain at below 0, indicating that the *bla*_CMY-2_ gene-harboring plasmid-free strain dominated. In *E. coli* J53 with the *bla*_CMY-2_ gene-harboring plasmid, a slight fitness decrease was observed, and the fitness was stable following continuous passage until 72 h. A greater reduction in fitness was observed in *E. coli* NCCP 13719 compared with *E. coli* J53 in the serial passage, with a reduction of more than 3 log units for lasting 72 h. Regarding the value of the log ratio of resistant versus susceptible strains, the *bla*_CMY-2_ gene-harboring plasmid imposed a slight fitness cost to *E. coli* J53 from about log −0.89 at 24 h to −0.97 at 72 h. In contrast, the transconjugant from *E. coli* NCCP 13719 showed quite a high fitness cost from about log −2.02 at 24 h to −5.58 at 72 h.

## 4. Discussion

Since the first finding of the CTX-M-type gene from Korea in 2001, the prevalence of the *bla*_CTX-M-15_-producing *Salmonella* in humans and chickens has rapidly increased over the years in Korea [3]. In the present study, the *bla*_CTX-M-15_ gene was the most frequently detected but showed low frequencies at <10^−7^ transconjugants per recipient, which is consistent with a previous study [21]. The *bla*_CTX-M-15_ genes belonging to IncFII plasmid are known to be highly prevalent and involved in the concurrent transfer of antimicrobial resistance and virulence genes, which increases co-selection and probably leads to the emergence or outbreaks of virulent and multidrug-resistant (MDR) clones [22].

Conversely, the *bla*_CMY-2_ gene was observed from one strain in this study. The first report of the *bla*_CMY-2_ gene was described in the 1990s [23], and now, it is one of the most common and widely disseminated genes by plasmid-mediated AmpC *β*-lactamase from humans and chickens [2]. Regarding the transfer frequency of the *bla*_CMY-2_ gene between bacteria, the results of this study explain how frequently their resistance gene gets transferred to other bacterial species. Frequent transfer of the IncI1 plasmid carrying the *bla*_CMY-2_ gene was measured with the ratio of over 10^−3^ transconjugants per recipient in this study. This result is higher than previous findings wherein the transfer frequencies of *bla*_CMY-2_ and *bla*_TEM-1_ genes from the *Salmonella* isolated from poultry meat were in the ratio of 6.0 × 10^−8^ to 2.4 × 10^−4^ transconjugants per recipient [24]. The high frequency of transfer and their possibility to exchange genes within and between species might have resulted in the increasing prevalence of the *bla*_CMY-2_ gene in animals and humans, and its rapid dissemination may constitute a significant risk to public health. To our knowledge, this is the first description of the transfer of a *bla*_CMY-2_ gene-harboring plasmid from chicken-origin *Salmonella enterica* to pathogenic *E. coli* isolated from a human patient in a mammalian model. Identifying the transfer of antimicrobial-resistant plasmids and their frequency in a mouse model, which is an adequate way to predict the risk of the dissemination of antimicrobial resistance genes with a perspective of food safety. From this point of view, we used a streptomycin-pretreated mouse model, which provides more realistic results than any in vitro or gnotobiotic study because the normal microflora barrier and the present immune system give the tested animal model advantages in mimicking the human gut [25]. In this study, *E. coli* transconjugant appeared in all mice fecal samples 1 dpi in G4, and the high transfer frequency observed with the mean ratio of transconjugants per recipient was about 2 × 10^−1^ and per donor was 4 × 10^−6^, which support statements on the rapid transfer of the *bla*_CMY-2_ gene. Although there is a lack of in vivo studies focused on the *bla*_CMY-2_ gene, several studies for conjugal transfer of ESBL genes have been reported. *bla*_TEM_ gene from *Salmonella* was transferred to *E. coli* recipient with the ratio of transconjugant per donor being 6.5 × 10^−5^ in mice without selective pressure [26], and *bla*_CTX-M-9_ gene derived from chicken-origin *Salmonella* to *E. coli* at a frequency range of about 5.4 × 10^−5^ in gnotobiotic rats [27]. It is important to emphasize that it demonstrated not only the capability of transfer of *bla*_CMY-2_ gene with high frequency but also showed that the ratio of transconjugants per recipient in vivo was 2 log units higher than in vitro. Similar findings were reported wherein the rate of plasmid transfer between *Enterococcus faecium* strains was up to 8 log units higher in the germ-free mice model than in vitro [28]. The high frequencies of plasmid transfer in vivo may be due to the constant mixing of bacteria by the peristaltic movements in the gastrointestinal tract, stimulating a donor with more access to recipients than during in vitro mating, wherein the bacterial movement is much lesser [29]. These results emphasized the necessity of in vivo test for transferability and transfer frequency to figure out the potential risk of the presence of resistant strains in the digestive tract to humans.

Our result could be a direct evidence that the ESC resistant *Salmonella* from chicken-related products can transfer their resistance gene to other pathogens, thus leading to the possibility of inappropriate antimicrobial selection and limited treatment options resulting in therapeutic failure [5]. A case of treatment failure due to the emergence of resistance to ceftriaxone, a 3rd generation of cephalosporin, has been reported. The originally susceptible pathogen developed ceftriaxone resistance via the acquisition of a plasmid containing the ceftriaxone resistance gene during the 3rd ESC treatment, which finally caused therapeutic failure in the patient [30]. In addition, even if resistant bacteria transiently colonize, it may quickly transfer resistance plasmid into the human gastrointestinal tract; normal microbiota and the nutrient-rich environment make the gastrointestinal tract offer an ideal condition for gene exchange [28]. In this study, two days after inoculation, about >10^4^ CFU/g of ceftiofur resistant *E. coli* isolates, regarded as normal flora-derived strain, were observed from one mouse in G2, which was inoculated only with *Salmonella* (donor). Likewise, the intestinal microbiota can act as a massive reservoir of antimicrobial resistance genes, thus prolonging the spread of MDR bacteria and resulting in therapeutic failure. Consequently, secondary infections would occur more often, indicating a serious threat to human health [31].

In vitro direct competition studies of the *bla*_CMY-2_ gene-harboring plasmid and two recipient *E. coli* showed that a variable fitness cost depends on the parent strains, and we observed that susceptible strains can outcompete resistant strains consistent with a previous study [17]. Normally, the acquisition of a plasmid often imposes a fitness burden on a bacterial cell [6]. Since *E. coli* NCCP 13719 in this study has the virulent gene *ipa*H, which may be encoded by a large plasmid, carrying another plasmid may present an adverse situation for the bacteria [6]. Conversely, the stable inheritance of bacterial plasmids without any selective pressure was also observed from transconjugants from *E. coli* J53 during the 72 h of experiment time. This phenomenon suggests that strains with low fitness costs even with the acquisition of plasmids from other strains may exist. For further studies, the mechanism of sustaining resistance plasmid with low fitness cost is expected to be a key research topic for suggesting the way to control the dissemination of antimicrobial resistance genes.

There are several limitations that we examined the characteristic of *bla*_CMY-2_ gene-harboring bacteria with a single strain; however, it may serve as fundamental data that defined the characteristics, and further studies with a greater number of resistant bacteria harboring the *bla*_CMY-2_ gene are required due to their increasing trend of emergence recently. In addition, mice are often naturally resistant to non-mice-origin *E. coli* colonization [32] as seen in our results, and thus, decreasing the number of bacteria is an inevitable phenomenon; however, it can be presented as a model that is sufficiently able to establish the transferability and frequency of antimicrobial resistance genes and emphasize that colonizing bacteria may transfer resistance plasmids readily in the intestinal tract [28]. To confirm the persistence of resistant genes through the colonization of antimicrobial-resistant bacteria in vivo and subsequent transfer of the gene to normal flora, changes in test strain or replacement of the in vivo model are required.

## 5. Conclusions

This study showed the prevalence of ESC resistance genes in *Salmonella* isolated from chickens and presented a comprehensive analysis of the highly frequent transfer of the *bla*_CMY-2_ gene in vitro and in vivo. In addition, this study has demonstrated the ease with which a *bla*_CMY-2_ gene-harboring plasmid can be rapidly transferred between *Salmonella* and pathogenic *E. coli* within the intestinal tracts of mice, even without antimicrobial selective pressure. Notably, we observed that once *bla*_CMY-2_ gene-harboring strains enter the mammalian intestinal tract, their dissemination could be more rapid and frequent than it would be in vitro, and even they could be transferred to the indigenous intestinal microbiota, threatening future treatments of infections. Since the use of cephalosporin in the poultry industry has increased over the last decade in Korea [33], the increasing emergence of ESBL/AmpC producing ESC resistant *Salmonella* spp. isolated from poultry is of concern. There is a risk for consumers related to exposure to ESBL/AmpC genes by contaminated food, so the application of guidelines for prudent antimicrobial usage in the poultry industry is urgently needed. Given the potential risk of the frequent transfer of the *bla*_CMY-2_ gene via the food chain to the human digestive tract, the molecular mechanism involved in the dissemination and maintenance of ESC resistance genes should be studied as further research in greater detail, and enhanced surveillance should be implemented to prevent the widespread of ESC resistant strains.

## Figures and Tables

**Table 1:** Information on 17 cephalosporin-resistant *Salmonella* spp. isolated from chicken-related sources.

No.	Strain	Serovar	Source	Antimicrobial Resistance	Phenotype	ESC Resistance Gene	Conjugation	Plasmid Type
1	A17-KCI-MNK-002-2S	S. Albany	Feces	AMC/AMP/FOX/TAZ/XNL/SXT/FIS/CHL/NAL/TET	AmpC	CMY-2	a	I1
2	A17-KCI-OP-004-4S	*S*. spp	Feces	AMP/TAZ/XNL/FEP/FIS/NAL/STR/TET	ESBL	CTX-M-15	NA	ND
3	A17-KCI-CRBR-001-5	*S*. Enteritidis	Carcass	AMP/TAZ/XNL/FEP/FIS/NAL/STR/GEN/TET	ESBL	CTX-M-15	b	FIIS
4	A17-KCI-HMR-001-4	*S.* Enteritidis	Carcass	AMP/XNL/SXT/FIS/CHL/CIP/NAL/STR/GEN/TET/COL	ESBL	TEM-1, CTX-M-15	NA	ND
5	A17-KCI-HMR-002-1	*S.* Enteritidis	Carcass	AMP/TAZ/XNL/FEP/NAL/GEN/TET	ESBL	CTX-M-15	NA	ND
6	A17-KCI-HMR-002-2	*S.* Enteritidis	Carcass	AMP/TAZ/XNL/FEP/NAL/GEN/TET	ESBL	CTX-M-15	NA	ND
7	A17-KCI-HMR-002-3	*S.* Enteritidis	Carcass	AMP/TAZ/XNL/FEP/NAL/GEN/TET	ESBL	CTX-M-15	b	FIIS
8	A17-KCI-HMR-002-4	*S.* Enteritidis	Carcass	AMP/TAZ/XNL/FEP/NAL/GEN/TET	ESBL	CTX-M-15	b	FIIS
9	A17-KCI-HMR-002-5	*S.* Enteritidis	Carcass	AMP/TAZ/XNL/FEP/NAL/GEN/TET	ESBL	CTX-M-15	NA	ND
10	A18-KCI-HMR-001-1S	*S.* Virchow	Feces	AMP/TAZ/XNL/FEP/FIS/NAL/STR/TET	ESBL	CTX-M-15	b	Nontypeable
11	A18-KCI-HMR-001-3S	*S.* Virchow	Feces	AMP/TAZ/XNL/FEP/SXT/FIS/CHL/NAL/STR/TET	ESBL	CTX-M-15	NA	ND
12	A18-KCI-HMR-001-4S	*S.* Virchow	Feces	AMP/TAZ/XNL/FEP/FIS/NAL/STR/TET	ESBL	CTX-M-15	b	Nontypeable
13	A18-KCI-HMR-002-2S	*S.* Virchow	Feces	AMP/TAZ/XNL/FEP/FIS/NAL/STR/TET	ESBL	CTX-M-15	b	Nontypeable
14	A18-KCI-HMR-002-3S	*S.* Virchow	Feces	AMP/TAZ/XNL/FEP/SXT/FIS/CHL/NAL/STR/TET	ESBL	CTX-M-15	NA	ND
15	A18-KCI-HMR-002-5S	*S.* Virchow	Feces	AMP/TAZ/XNL/FEP/SXT/FIS/CHL/NAL/STR/TET	ESBL	CTX-M-15	NA	ND
16	A18-KCI-OP-003-2S	*S.* Enteritidis	Feces	AMP/TAZ/XNL/FEP/NAL/TET	ESBL	CTX-M-15	NA	ND
17	A18-KCI-OP-003-3S	*S*. Enteritidis	Feces	AMP/TAZ/XNL/FEP/NAL/TET	ESBL	CTX-M-15	NA	ND

AMC, Amoxicillin/clavulanic acid; AMP, Ampicillin; FOX, cefoxitin; TAZ, Ceftazidime; XNL, Ceftiofur; FEP, cefepime; SXT, Trimethoprim/sulfamethoxazole; FIS, sulfisoxazole; CHL, chloramphenicol; CIP, ciprofloxacin; NAL, nalidixic acid; STR, streptomycin; GEN, gentamicin; TET, tetracycline; COL, colistin. a, Conjugation frequency (Transconjugant/recipient) with ≥10^−3^; b, Conjugation frequency with <10^−7^. NA, not available; ND, not done.

**Table 2:** Characteristic changes of transconjugant after conjugation and transfer frequency of the resistance gene.

Strain ^a^		Species	MIC (μg/mL)							Phenotype	ESC Resistance Gene	Conjugation Frequency ^b^
			FOX	XNL	TAZ	FEP	AMP	AMC	TET			
			≥32	≥8	≥16	≥16	≥32	≥32/16	≥16			
MNK	Donor	*S.* Albany	>32	>8	>16	1	>64	>32/16	32	AmpC	CMY-2	
J53	Recipient	*E. coli*	8	<0.5	<1	<0.25	< 2	4/2	<2			
Tc.J53	Transconjugant		>32	>8	>16	<0.25	>64	>32/16	<2	AmpC	CMY-2	1 × 10^−3^ ± 1 × 10^−7^
13719 (EIEC)	Recipient	*E. coli*	8	<0.5	<1	<0.25	8	4/2	>128			
Tc.13719	Transconjugant		>32	>8	>16	<0.25	>64	>32/16	>128	AmpC	CMY-2	2 × 10^−3^ ± 1 × 10^−3^

FOX, cefoxitin; XNL, ceftiofur; TAZ, ceftazidime; FEP, cefepime; AMP, ampicillin; AMC, amoxicillin/clavulanic acid; TET, tetracycline. ^a^ MNK, *Salmonella* Albany A17-KCI-MNK-002-2S; J53, *Escherichia coli* J53; Tc.J53, transconjugant of *E. coli* J53; 13719, *E. coli* NCCP 13719; Tc.13719, transconjugant of *E. coli* NCCP 13719; EIEC, enteroinvasive *Escherichia coli*. ^b^ Transconjugant per recipient (Tc/R).

## Data Availability

The data presented in this study are available from the corresponding author on reasonable request.

## Supplementary Materials

The following are available online at: https://www.mdpi.com/article/10.3390/ani11061778/s1. Table S1. The list of pathogenic *Escherichia coli* isolates was obtained from the National Culture Collection for Pathogen (NCCP), based in Korea, Figure S1. Fecal excretion of the donor in group 2 (G2) and the recipient in group 3 (G3) (a), the donor, recipient, and transconjugant in group 4 (G4) (inoculation of donor and recipient, simultaneously) (b), expressed as the log number of CFU per gram of feces.
