# Supplementary material for: Characterization of Extended-Spectrum Cephalosporin (ESC) Resistance in Salmonella Isolated from Chicken and Identification of High Frequency Transfer of blaCMY-2 Gene Harboring Plasmid In Vitro and In Vivo
Source: Animals (Basel). 2021 Jun 14;11(6):1778. doi: 10.3390/ani11061778 (PMC8232285; doi:10.3390/ani11061778)
Supplement: Supplementary file 1 [file animals-11-01778-s001.zip › animals-1226303-supplementary.pdf]

cancers

MDPI

Supplementary Materials

Characterization of Extended-Spectrum Cephalosporin (ESC) Resistance in *Salmonella* Isolated from Chicken and Identification of High Frequency Transfer of *bla*$_{CMY-2}$ Gene Harboring Plasmid In Vitro and In Vivo

Bo-Ram Kwon, Bai Wei, Se-yeoun Cha, Ke Shang, Jun-Feng Zhang, Hyung-Kwan Jang and Min Kang

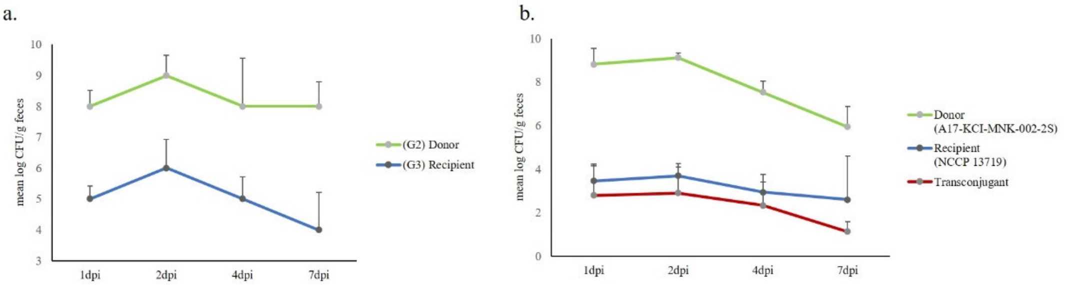

Figure S1. Fecal excretion of the donor in group 2 (G2) and the recipient in group 3 (G3) (a), the donor, re-cipient, and transconjugant in group 4 (G4) (inoculation of donor and recipient, simultaneously) (b), expressed as the log number of CFU per gram of feces.

Table S1. The list of pathogenic Escherichia coli isolates obtained from the National Culture Collection for Pathogen (NCCP) based in Korea.

No.	NCCP no.	Pathogenic	Antimicrobial resistance	Conjugation
1	14039	EAEC	AMP/SXT/FIS/STR/TET	b
2	15649	EAEC	AMP/SXT/FIS/CHL/STR/TET	b
3	13719	EIEC	FIS/TET	a
4	15663	EIEC	SXT/FIS/STR	b
5	15739	EHEC	AMP/STR/FIS	b
6	15954	EHEC	AMP/STR/FIS	b
7	15732	ETEC	Susceptible	-*
8	15741	ETEC	Susceptible	-
9	14038	EPEC	Susceptible	-
10	15661	EPEC	Susceptible	-

AMP, Ampicillin; SXT, Trimethoprim/sulfamethoxazole; FIS, sulfisoxazole; CHL, chloramphenicol; STR, streptomycin; TET, tetracycline.
a, Conjugation frequency (Transconjugant/recipient) with ⩾10-3; b, Conjugation frequency with <10-7.
*Antimicrobial-susceptible isolates could not be used for conjugation experiment.
